# Multimorbidity and cancer treatment among the older patients in the United States

**DOI:** 10.1371/journal.pone.0338721

**Published:** 2026-01-07

**Authors:** Tung Thanh Pham, Avonne E. Connor, Anne F. Rositch

**Affiliations:** 1 College of Health Sciences, VinUniversity, Hanoi, Vietnam; 2 Department of Epidemiology, Harvard T.H. Chan School of Public Health, Boston, Massachusetts, United States of America; 3 Research Advancement Consortium in Health (REACH), Hanoi, Vietnam; 4 Department of Epidemiology, Johns Hopkins Bloomberg School of Public Health, Baltimore, Maryland, United States of America; 5 Department of Oncology, Johns Hopkins Sidney Kimmel Comprehensive Cancer Center, Baltimore, Maryland, United States of America; 6 Health Outcomes and Real World Evidence, Hologic, Inc, Marlborough, Massachusetts, United States of America; Luxembourg Institute of Health, LUXEMBOURG

## Abstract

**Introduction:**

The number of individuals who are diagnosed with cancer and other comorbidities continues to increase, and the average number of comorbidities among racial/ethnic minority patients is higher than non-Hispanic (N.H.)-white patients. Therefore, we explored the association between race/ethnicity, comorbidities, and cancer treatment among older Americans diagnosed with the four most common cancer types.

**Methods:**

In this retrospective cohort study, SEER-Medicare linked data were used to identify 692,159 individuals over 65 years old diagnosed with female breast, colorectal, lung, or prostate cancer from 1992–2011. Multimorbidity was defined as having cancer plus two or more comorbidities. Modified Poisson regression models were used to assess the association between comorbidities and race/ethnicity on cancer treatment within 6 months of diagnosis.

**Results:**

For all cancers, the percentage of patients receiving treatment declined over time and with increasing age, number of comorbidities, and advanced cancer stage. Variability in receipt of treatment by race/ethnicity was observed: 76% for NH-White, 75% for Hispanic, and 68% for NH-Black patients. Concurrently, multimorbidity was increasing over time for all patients. Adjusting for other covariates, patients with multimorbidity were less likely to receive cancer treatment (RR = 0.987–0.947, all p-value<0.001). Moreover, NH-Black (RR = 0.955–0.865, all p-value<0.001) and Hispanic (RR = 0.987–0.951, all p-value<0.001) patients without multimorbidity were less likely to be treated compared to NH-White patients without multimorbidity.

**Conclusions:**

Our findings suggest that the prevalence of multimorbidity among older patients with cancer has increased and negatively affected cancer treatment among this population. Racial disparities may exist in cancer treatment and seem to be more pronounced in patients without multimorbidity.

## Introduction

In 2019, approximately one in every six individuals (around 54 million) in the United States was over 65 years old [1]. This population will grow to nearly 84 million by 2050 [2]. Life expectancy at age 65 is also dramatically increasing, from 15 years in the 1970s to nearly 20 years in 2018 [2,3]. As the proportion of this older population increases, the prevalence of chronic diseases, especially cancer, increases rapidly. More than half of new cancer cases are diagnosed in patients older than 65 years old [4].

Over 65% of older patients with cancer also have multiple medical comorbidities [5,6]. The disease management process for these patients can become increasingly complicated as physicians manage multiple combinations of drugs and therapies [7,8]. Patients with advanced age are also at risk of increased toxicity, lower treatment efficacy, and tolerance [9,10]; their participation in clinical trials, especially older patients with cancer and with comorbidities, has been limited [9,11–13]. Therefore, the lack of evidence makes it difficult for physicians to make treatment recommendations when considering the risks and benefits of complex treatment regimens. In this case, a careful assessment of comorbidities, health, and functional status may be needed to avoid any intervention that would negatively affect the quality of life and have only modest benefits [13–18].

To further complicate this issue, racial/ethnic minority patients with cancer are more likely to have multiple comorbidities than white patients [19,20]. This disparity could adversely affect the receipt of cancer treatment among these minority patient groups. Therefore, we aim to describe the prevalence and patterns of multimorbidity and explore the association between race/ethnicity, multimorbidity, and cancer treatment among older Americans diagnosed with the four most common cancer types: female breast, colorectal, lung, and prostate.

## Methods

### Study population and design

This study was a retrospective cohort of older patients with cancer in the United States in the Surveillance, Epidemiology, and End Results (SEER)-Medicare linkage database [21]. Individuals were included if they were (1) White, Black, or Hispanic individuals; (2) Diagnosed with breast, colorectal, lung, and prostate cancers; (3) Diagnosed at age 66 or older so that there was at least one year of data available to explore the diagnosis of multimorbidity prior to cancer diagnosis and treatment; (4) Diagnosed with cancer on/after 01/1992 and before 12/2011 so that at least one year of follow-up after the diagnosis is available in both the SEER and Medicare data (data through 12/2012); and (5) With Medicare part A, part B, and non-HMO continuous coverage from at least one year before to at least one year after the date of a cancer diagnosis. This study was deemed exempt by the Institutional Review Board at Johns Hopkins Bloomberg School of Public Health.

The primary study outcome, receipt of initial cancer treatment, was defined as receiving any cancer-type-specific surgery, chemotherapy including oral prescriptions, radiotherapy, hormone, transplant, immune therapy, or any combination of these therapies within six months of a cancer diagnosis. We ascertained these outcomes using Medicare claims filed for treatment services after the cancer diagnosis. We examined the National Claims History (NCH), Outpatient, Medicare Provider Analysis and Review (MEDPAR), and durable medical equipment (DME) data files to identify cancer-type-specific treatments. In particular, we searched these records for codes that indicate treatment that is standard to cure or contribute to curing each cancer type. These codes were then compared against the National Comprehensive Cancer Network Guidelines to reduce non-specific codes and were reviewed by an oncologist with expertise in using large databases to examine cancer treatment, as previously described in other papers [22–25]. There could be overlap between curative intent and palliative intent treatment in these treatment codes, and we provided the full code list in a previous paper for future replication studies [23].

The extent of comorbidities was measured in two ways: first, using the list of conditions from a modified version of the Charlson Comorbidity Index (CCI) [26] developed by Quan and colleagues [27], and then second by creating a binary variable for multimorbidity. We used the Medicare claims to identify the diagnosis of 17 comorbidities in the CCI, and these diagnoses had to appear in at least two different claims that were more than 30 days apart before the cancer diagnosis [28]. In calculating CCI and the number of comorbidities for this paper, we excluded cancer and cancer metastasis to avoid collinearity with other variables, such as cancer stage. Multimorbidity was defined as having cancer plus two or more comorbidities included in the CCI [29,30].

Other available covariates of interest included in the analysis were age, sex, race/ethnicity, year of cancer diagnosis, cancer stage at diagnosis, and socioeconomic status (SES). For SES, we created a composite binary variable based on zip code level median income, percent of high-school graduates, and percent of residents living below poverty to avoid collinearity between these variables [22,23]. A participant is considered to have an indicator of lower SES when residing in an area where: (1) the median income is less than the median income of all available areas; (2) the percentage of non–high school graduates is greater than the median percentage of non–high school grads of all available areas; or (3) the percentage of residents living below the poverty line is greater than the median percentage of residents living below the poverty level of all available areas. Participants with missing zip code level SES information for all three variables were excluded from our analysis (1.8%, n = 12,413).

### Statistical analysis

In this study, all categorical variables were presented as the number of participants and percentage, and continuous variables were presented as median and interquartile range (IQR) or mean and standard deviation (S.D.) depending on the distribution of the variable. To extract the trend of multimorbidity and cancer treatment, we plotted the proportion of patients receiving cancer treatment and the proportion of patients with multimorbidity over time, as well as treatment percentage vs. the number of morbidities. We also used the National Cancer Institute’s Joinpoint Trend Analysis Software Version 4.9.1.0 to evaluate significant multimorbidity and cancer treatment changes over time [31].

Since the prevalence of receiving cancer treatment was high among our participants, Poisson regression models with robust estimates of variance were used to calculate relative risks (RRs) with similar results compared to log-binomial models, which often result in nonconvergence issues [32–34]. All of these Poisson regression models were cancer-type-specific and adjusted for other covariates (race/ethnicity, cancer stage, composite SES, year of diagnosis, and gender when appropriate). Potential statistical interaction was explored by introducing an interaction term for multimorbidity indicator or CCI score and race/ethnicity and stratifying on multimorbidity/race. All analyses were conducted using Stata Version 17.0.

## Results

In this study, the median age of the 692,159 patients was 75 (IQR: 70–81). Most of the participants were Non-Hispanic White (NHW) patients (86%) followed by Non-Hispanic Black (NHB) (9%) and Hispanic patients (5%; **Table 1**). Among all participants, prostate cancer accounted for 32% of cancer cases, while lung cancer was the next most common (29%), then colorectal (20%) and breast cancer (19%). Although this order still applied among males (prostate: 57%; lung: 27%; colorectal: 16%), breast cancer was the most common cancer among females 43% followed by lung: (32%) and colorectal cancer (25%). Overall, 22% of patients were diagnosed with distant-stage cancer, with great variability by cancer type (lung: 51%; colorectal: 20%; breast: 7%; prostate: 5%).

**Table 1:** Characteristic of participants.

	Breast	Colorectal	Lung	Prostate	Total
	N = 131,888	N = 137,414	N = 203,502	N = 219,355	N = 692,159
Age group					
65–70	32,515 (25%)	25,156 (18%)	50,093 (25%)	68,620 (31%)	176,384 (25%)
71–75	31,766 (24%)	29,560 (22%)	54,194 (27%)	65,697 (30%)	181,217 (26%)
76–80	29,099 (22%)	30,704 (22%)	48,279 (24%)	46,251 (21%)	154,333 (22%)
81 or above	38,508 (29%)	51,994 (38%)	50,936 (25%)	38,787 (18%)	180,225 (26%)
Gender					
Female	131,888 (100%)	77,447 (56%)	98,007 (48%)	0 (0%)	307,342 (44%)
Male	0 (0%)	59,967 (44%)	105,495 (52%)	219,355 (100%)	384,817 (56%)
Race/Ethnicity					
Non-Hispanic white	115,850 (88%)	118,216 (86%)	178,047 (87%)	180,749 (82%)	592,862 (86%)
Non-Hispanic black	10,221 (8%)	12,154 (9%)	17,623 (9%)	25,406 (12%)	65,404 (9%)
Hispanic	5,817 (4%)	7,044 (5%)	7,832 (4%)	13,200 (6%)	33,893 (5%)
Year of Cancer Diagnosis					
1991-1995	17,486 (13%)	20,287 (15%)	25,918 (13%)	35,272 (16%)	98,963 (14%)
1996-2000	20,405 (15%)	22,839 (17%)	29,097 (14%)	32,083 (15%)	104,424 (15%)
2001-2005	42,614 (32%)	47,262 (34%)	69,492 (34%)	70,911 (32%)	230,279 (33%)
2006-2011	51,383 (39%)	47,026 (34%)	78,995 (39%)	81,089 (37%)	258,493 (37%)
Indicator of lower SES					
No	89,476 (68%)	88,418 (64%)	124,298 (61%)	145,133 (66%)	447,325 (65%)
Yes	42,412 (32%)	48,996 (36%)	79,204 (39%)	74,222 (34%)	244,834 (35%)
Cancer stage					
Localized/Regional	118,014 (89%)	101,149 (74%)	81,524 (40%)	170,589 (78%)	471,276 (68%)
Distant	9,767 (7%)	27,513 (20%)	104,736 (51%)	10,912 (5%)	152,928 (22%)
Unknown	4,107 (3%)	8,752 (6%)	17,242 (8%)	37,854 (17%)	67,955 (10%)
Ever treatment within 6 months					
No	10,544 (8%)	20,406 (15%)	75,587 (37%)	62,150 (28%)	168,687 (24%)
Yes	121,344 (92%)	117,008 (85%)	127,915 (63%)	157,205 (72%)	523,472 (76%)
Number of comorbidities					
1	58,035 (44%)	50,586 (37%)	49,777 (24%)	99,765 (45%)	258,163 (37%)
2	37,252 (28%)	36,803 (27%)	56,192 (28%)	62,199 (28%)	192,446 (28%)
3	19,223 (15%)	22,979 (17%)	42,158 (21%)	30,750 (14%)	115,110 (17%)
4+	17,378 (13%)	27,046 (20%)	55,375 (27%)	26,641 (12%)	126,440 (18%)
Number of comorbidities	2.00 (1.00-3.00)	2.00 (1.00-3.00)	2.00 (2.00-4.00)	2.00 (1.00-3.00)	2.00 (1.00-3.00)

Data are Presented as median (interquartile range) for continuous measures, and n (%) for categorical measures.

Nearly two-thirds of patients had two or more comorbidities besides their cancer and met the definition of multimorbidity (62.70%); nearly 1 in 5 patients (18.27%) had four or more comorbidities. We also observed that lung and colorectal patients were more likely to have multimorbidity than breast and prostate cancer patients (76% & 63% vs. 56% & 55%, respectively). Among all patients with cancer, the most common comorbidities were chronic pulmonary disease (29.45%), diabetes without chronic complication (18.48%), and peripheral vascular disease (16.93%) **(Table A in** **S1 File**). This trend was consistent in breast, colorectal, and prostate patients with cancer. Among lung patients with cancer, chronic pulmonary disease (51.27%) was still the most common comorbidity, followed by peripheral vascular disease (23.34%) and congestive heart failure (21.33%).

We observed that the receipt of treatment overall declined with increasing age, number of comorbidities, and advanced cancer stage (**Table 2**). The proportion of individuals who received therapies was highest for breast cancer (92.01%), followed by colorectal (85.15%), prostate (71.67%), and lung cancer (62.86%). Moreover, variability in receipt of treatment by race/ethnicity was observed: 76.44% for NHW patients, 75.18% for Hispanic patients, and 68.47% for NHB patients (global p-value < 0.001).

**Table 2:** The receipt of cancer treatment among all participants.

Number treated (%)	Breast	Colorectal	Lung	Prostate	Total
	121,344 (92%)	117,008 (85%)	127,915 (63%)	157,205 (72%)	523,472 (76%)
Age group					
65–70	31,189 (96%)	22,655 (90%)	37,785 (75%)	53,592 (78%)	145,221 (82%)
71–75	30,259 (95%)	26,580 (90%)	38,329 (71%)	49,452 (75%)	144,620 (80%)
76–80	27,267 (94%)	27,008 (88%)	30,380 (63%)	31,552 (68%)	116,207 (75%)
81 or above	32,629 (85%)	40,765 (78%)	21,421 (42%)	22,609 (58%)	117,424 (65%)
Gender					
Female	121,344 (92%)	66,134 (85%)	61,252 (62%)	N/A	248,730 (81%)
Male	N/A	50,874 (85%)	66,663 (63%)	157,205 (72%)	274,742 (71%)
Race/Ethnicity					
Non-Hispanic white	107,217 (93%)	101,417 (86%)	113,172 (64%)	131,404 (73%)	453,210 (76%)
Non-Hispanic black	8,818 (86%)	9,622 (79%)	10,028 (57%)	16,312 (64%)	44,780 (68%)
Hispanic	5,309 (91%)	5,969 (85%)	4,715 (60%)	9,489 (72%)	25,482 (75%)
Year of Cancer Diagnosis					
1991-1995	16,369 (94%)	17,654 (87%)	16,741 (65%)	23,757 (67%)	74,521 (75%)
1996-2000	19,014 (93%)	19,853 (87%)	18,496 (64%)	22,529 (70%)	79,892 (77%)
2001-2005	39,213 (92%)	41,120 (87%)	43,433 (63%)	53,113 (75%)	176,879 (77%)
2006-2011	46,748 (91%)	38,381 (82%)	49,245 (62%)	57,806 (71%)	192,180 (74%)
Indicator of lower SES					
No	82,749 (92%)	75,917 (86%)	80,429 (65%)	105,573 (73%)	344,668 (77%)
Yes	38,595 (91%)	41,091 (84%)	47,486 (60%)	51,632 (70%)	178,804 (73%)
Cancer stage					
Localized/Regional	113,321 (96%)	93,357 (92%)	61,206 (75%)	125,150 (73%)	393,034 (83%)
Distant	6,801 (70%)	20,345 (74%)	61,930 (59%)	8,079 (74%)	97,155 (64%)
Unknown	1,222 (30%)	3,306 (38%)	4,779 (28%)	23,976 (63%)	33,283 (49%)
Number of comorbidities					
1	53,934 (93%)	43,506 (86%)	31,569 (63%)	71,692 (72%)	200,701 (78%)
2	34,940 (94%)	32,293 (88%)	38,032 (68%)	46,227 (74%)	151,492 (79%)
3	17,502 (91%)	19,514 (85%)	27,039 (64%)	21,879 (71%)	85,934 (75%)
4+	14,968 (86%)	21,695 (80%)	31,275 (56%)	17,407 (65%)	85,345 (67%)

**Fig 1:**
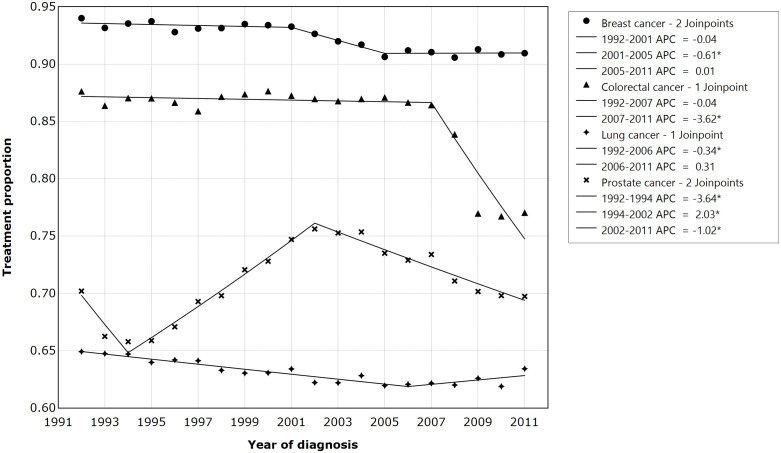
The statistical trends of cancer treatment over time with join points and annual percent change (APC). APC: Annual percent change of each period (%). * indicated statiiscical significant APC change within a period. Joinpoints indicated the number of joinpoints selected by the Joinpoint statistical program.

Over time, the percentage of patients who received treatment decreased among breast, colorectal, and lung patients, while it fluctuated for prostate patients with cancer. The Annual Percent Change (APC) in treatment decreased significantly among patients with breast (APC from 2001–2005 = −0.61%), colorectal (APC 2007–2011 = −3.62%), lung (APC 1992–2006 = −0.34%), and prostate cancer (APC 1992–1994 = −3.64%; APC 1994–2002 = 2.03%; APC 2002–2011 = −1.02%) (**Fig 1**). Concurrently, the proportion of patients with multimorbidity increased rapidly over time regardless of cancer type **(Figure A and B in** S1 File**)**. The APC in multimorbidity increased significantly among patients diagnosed with breast (APC 1992–1996 = 4.44%; APC 1996–2011 = 2.12%), colorectal (APC 1992–1998 = 4.32%; APC 1998–2004 = 1.84%; APC 2004–2008 = 3.23%), lung (APC 1992–2001 = 3.32%; APC 2001–2011 = 2.17%), and prostate cancer (APC 1992–2011 = 1.95%) **(Figure A in** S1 File**)**.

**Fig 2:**
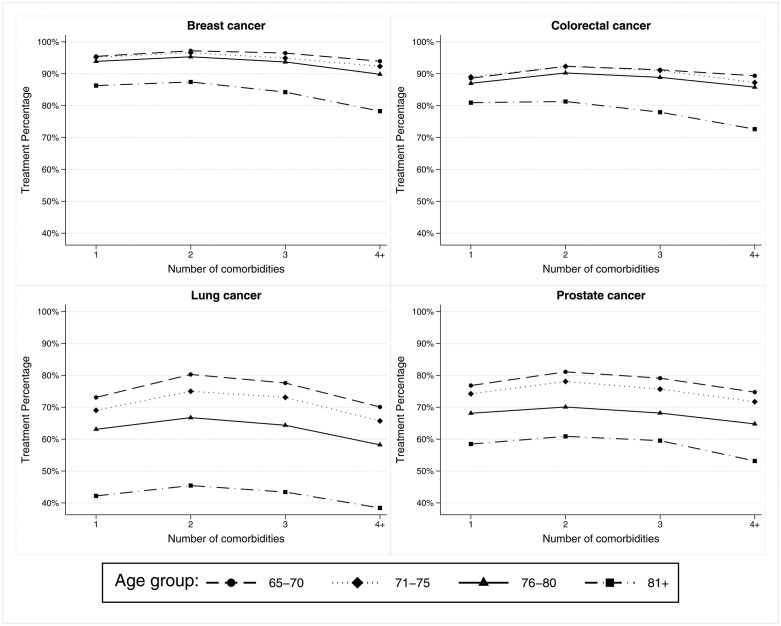
The receipt of cancer treatment and number of comorbidities by age groups.

There was a modest increase in treatment percentage when comparing patients with one comorbidity (without multimorbidity) to patients with two comorbidities (with multimorbidity) **(Fig 2**). However, within each age group, the treatment percentage declined gradually as comorbidities increased. However, the treatment percentage was uniformly lower among the older age groups than younger ages **(Fig 2)**. For example, among breast and colorectal patients with cancer, the treatment patterns were consistent among individuals aged 65–70, 71–75, and 76–80, but the treatment percentage was 7–10% lower among individuals aged 81 and above compared to the younger group.

**Fig 3:**
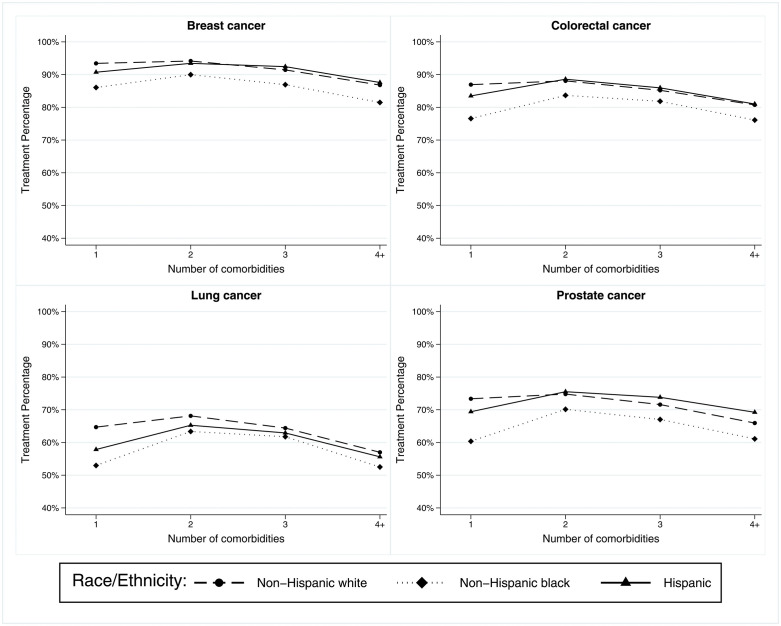
The receipt of cancer treatment and number of comorbidities by race/ethnicity.

**Fig 3** demonstrated a clear separation of treatment patterns by age groups among lung and prostate patients with cancer; the differences between the youngest and oldest age groups could be up to 30%. We also observed that the treatment percentage was consistently lower for NHB patients than NHW and Hispanic patients (**Fig 3**). Interestingly, among breast and colorectal cancer patients, NHW and Hispanic patients’ treatment patterns were comparable, while NHB patients’ treatment pattern was lower by 5–7% compared to the other group. Moreover, the racial differences in treatment patterns were less noticeable in lung cancer compared to prostate cancer.

Overall, patients with cancer with multimorbidity were less likely to receive cancer treatment (unadjusted RR = 0.910, 0.950, 0.951, and 0.940 for lung, colorectal, breast, and prostate cancer, respectively, all p-value < 0.001) **(Table B in** S1 File**)**. In the unadjusted models for all cancer types, the likelihood of cancer treatment was also shown to be consistently lower among those individuals with older age and later diagnosis date, NHB- and Hispanic patients, those with late stage at diagnosis, and lower socioeconomic status **(Table B in** S1 File**).** After adjusting for other covariates, the effect of multimorbidity was attenuated, but patients with multimorbidity were still less likely to receive treatment compared to patients without multimorbidity regardless of cancer type (adjusted RR = 0.987 to 0.947, all p-value < 0.001) (**Table 3**). Lung patients with cancer with multimorbidity experienced the largest relative decrease in likelihood of treatment compared to those without multimorbidity (adjusted RR = 0.947, p-value < 0.001) while colorectal, breast, and prostate patients with cancer with multimorbidity experience relatively similar decreases (adjusted RR = 0.987, 0.982, and 0.979, respectively, all p-value < 0.001). When using CCI score rather than multimorbidity indicator in the multivariable regression models, the results were still consistent that treatment proportion decreased with higher CCI score **(Table C in** S1 File**).**

**Table 3:** Multivariable regression model for the receipt of cancer treatment using multimorbidity indicator.

VARIABLES	Breast			Colorectal			Lung			Prostate		
	RR	95% CI	p-value	RR	95% CI	p-value	RR	95% CI	p-value	RR	95% CI	p-value
Age group												
65–70	REF			REF			REF			REF		
71–75	0.996	0.993 - 0.999	0.008	0.999	0.993 - 1.004	0.606	0.941	0.935 - 0.948	<0.001	0.964	0.959 - 0.970	<0.001
76–80	0.984	0.981 - 0.987	<0.001	0.980	0.974 - 0.985	<0.001	0.846	0.839 - 0.853	<0.001	0.874	0.867 - 0.880	<0.001
81 or above	0.922	0.918 - 0.926	<0.001	0.902	0.897 - 0.908	<0.001	0.589	0.583 - 0.596	<0.001	0.749	0.741 - 0.756	<0.001
Gender												
Female				REF			REF					
Male				0.975	0.971 - 0.979	<0.001	0.990	0.984 - 0.996	0.002			
Race/Ethnicity												
Non-Hispanic white	REF			REF			REF			REF		
Non-Hispanic black	0.952	0.946 - 0.959	<0.001	0.941	0.933 - 0.949	<0.001	0.903	0.892 - 0.915	<0.001	0.879	0.870 - 0.888	<0.001
Hispanic	0.982	0.975 - 0.989	<0.001	0.989	0.979 - 0.998	0.020	0.970	0.954 - 0.987	0.001	0.984	0.973 - 0.995	0.005
Year of Cancer Diagnosis												
1991-1995	REF			REF			REF					
1996-2000	0.995	0.990 - 0.999	0.020	0.998	0.992 - 1.005	0.642	0.993	0.981 - 1.004	0.207	0.920	0.907 - 0.932	<0.001
2001-2005	0.982	0.978 - 0.986	<0.001	0.997	0.992 - 1.003	0.352	0.988	0.978 - 0.998	0.015	0.980	0.967 - 0.992	0.002
2006-2011	0.967	0.963 - 0.970	<0.001	0.936	0.930 - 0.942	<0.001	0.992	0.982 - 1.002	0.099	0.924	0.912 - 0.936	<0.001
Indicator of lower SES												
No	REF			REF			REF			REF		
Yes	1.001	0.998 - 1.004	0.422	0.992	0.988 - 0.997	0.001	0.926	0.920 - 0.932	<0.001	0.979	0.973 - 0.985	<0.001
Cancer stage												
Localized/Regional	REF			REF			REF			REF		
Distant	0.730	0.721 - 0.740	<0.001	0.801	0.796 - 0.807	<0.001	0.795	0.790 - 0.800	<0.001	1.089	1.076 - 1.101	<0.001
Unknown	0.318	0.304 - 0.334	<0.001	0.420	0.409 - 0.432	<0.001	0.405	0.395 - 0.414	<0.001	0.850	0.839 - 0.861	<0.001
Multimorbidity												
No	REF			REF			REF			REF		
Yes	0.982	0.978 - 0.985	<0.001	0.987	0.983 - 0.991	<0.001	0.947	0.941 - 0.953	<0.001	0.979	0.973 - 0.985	<0.001
Observations	131,888			137,414			203,502			219,355		

All R.R.s are fully adjusted from the full model with age groups, gender (if applicable), race/ethnicity, year of cancer diagnosis, indicator of lower SES, cancer stage, and multimorbidity.

Using stratified multivariable models by multimorbidity indicator and race **(Table 4)**, we found that, within the no multimorbidity group, NHB (adjusted RR = 0.955 to 0.865, all p-value < 0.001) and Hispanic (adjusted RR = 0.987 to 0.951, all p-value < 0.001) patients were less likely to be treated compared to NHW patients. However, the magnitude of differences in treatment status was smaller among patients with multimorbidity. This disparity was most pronounced in prostate cancer, followed by lung, and colorectal (all p-values for interaction < 0.001) compared to breast cancer (all p-values for interaction > 0.05). When stratified by race, multimorbidity is associated with decreased treatment in all cancer for NHW (adjusted RR = 0.940 to 0.983, all p-value < 0.001) and only breast cancer for NHB (adjusted RR = 0.978, p-value < 0.001).”

**Table 4:** Stratified models by multimorbidity indicator and race in the receipt of cancer treatment.

VARIABLES	Breast			Colorectal			Lung			Prostate		
	RR	95% CI	p-value	RR	95% CI	p-value	RR	95% CI	p-value	RR	95% CI	p-value
Interaction terms for multimorbidity indicator and race												
Non-Hispanic black*multimorbidity	1.000	0.975 - 1.004	0.157	**1.036**	**1.018 - 1.054**	**<0.001**	**1.062**	**1.035 - 1.089**	**<0.001**	**1.051**	**1.030 - 1.073**	**<0.001**
Hispanic*multimorbidity	1.014	1.011 - 1.019	0.089	1.012	0.993 - 1.032	0.223	**1.044**	**1.009 - 1.081**	**0.014**	**1.062**	**1.037 - 1.088**	**<0.001**
Stratified models by multimorbidity indicator												
No												
Race/Ethnicity												
Non-Hispanic white	REF			REF			REF			REF		
Non-Hispanic black	**0.955**	**0.947 - 0.964**	**<0.001**	**0.926**	**0.915 - 0.938**	**<0.001**	**0.878**	**0.862 - 0.894**	**<0.001**	**0.865**	**0.855 - 0.875**	**<0.001**
Hispanic	**0.978**	**0.969 - 0.986**	**<0.001**	**0.987**	**0.975 - 0.999**	**0.029**	**0.951**	**0.928 - 0.974**	**<0.001**	**0.968**	**0.955 - 0.981**	**<0.001**
Yes												
Race/Ethnicity												
Non-Hispanic white	REF			REF			REF			REF		
Non-Hispanic black	**0.947**	**0.935 - 0.958**	**<0.001**	**0.963**	**0.950 - 0.976**	**<0.001**	**0.933**	**0.916 - 0.950**	**<0.001**	**0.911**	**0.895 - 0.927**	**<0.001**
Hispanic	0.989	0.975 - 1.002	0.104	0.993	0.977 - 1.008	0.355	0.993	0.969 - 1.019	0.600	**1.025**	**1.004 - 1.046**	**0.018**
Stratified models by Race/Ethnicity												
Non-Hispanic white												
Multimorbidity												
No	REF			REF			REF			REF		
Yes	**0.981**	**0.977 - 0.985**	**<0.001**	**0.983**	**0.979 - 0.988**	**<0.001**	**0.940**	**0.934 - 0.946**	**<0.001**	**0.970**	**0.964 - 0.977**	**<0.001**
Non-Hispanic black												
Multimorbidity												
No	REF			REF			REF			REF		
Yes	**0.978**	**0.964 - 0.992**	**0.002**	**1.019**	**1.002 - 1.036**	**0.027**	0.995	0.971 - 1.020	0.716	1.014	0.994 - 1.033	0.170
Hispanic												
Multimorbidity												
No	REF			REF			REF			REF		
Yes	0.997	0.982 - 1.013	0.743	0.992	0.974 - 1.012	0.438	0.990	0.956 - 1.025	0.563	1.017	0.994 - 1.041	0.154

All RRs are fully adjusted for age groups, gender (if applicable), year of cancer diagnosis, indicator of lower SES, and cancer stage.

Bold indicated statistically significant with p-value < 0.05.

CCI score was calculated without cancer weight.

## Discussion

Using the SEER-Medicare linked data, we found that nearly two-thirds of patients with common cancers in the United States have multimorbidity. The proportion of patients with multimorbidity is higher among lung and colorectal cancer compared to breast and prostate cancer. This result is expected since lung cancer is closely related to smoking, which is a major risk factor for many chronic diseases, and colorectal cancer is associated with many lifestyle risk factors [35,36]. Due to the aging and increased prevalence of risk factors, such as obesity [37], in the U.S. population, multimorbidity has been increasing over time, and we could expect that this number will continue to increase in the future. Moreover, patients with multimorbidity were less likely to receive cancer treatment, and there were racial disparities in treatment status regardless of multimorbidity. These findings suggest that multimorbidity has negatively affected the receipt of cancer treatment among the older population compared to the population without multimorbidity.

Regarding the trend of cancer treatment, we observed that older age and advanced cancer stage are associated with lower treatment among all patients. This trend is unsurprising because curative therapies, as we tried to define using treatment code, have shown to be less beneficial with these factors and could negatively affect patients’ quality of life tolerance [9,10]. Regarding this trend over time, the percentage of treated patients significantly decreased for breast, colorectal, and lung cancer while it fluctuated for prostate cancer. The specific increase in prostate cancer treatment in 1994–2002 could be attributable to the approval of prostate-specific antigen (PSA) screening for prostate cancer in 1994 [38–40]. However, in 2002, the U.S. Preventive Services Task Force did not find sufficient evidence to recommend for or against PSA screening, and this fact may contribute to the decline in prostate cancer treatment in 2002–2011 [41].

We found that patients with cancer with multimorbidity were less likely to receive cancer treatment, independent of other factors such as race/ethnicity, cancer stage, SES, year of diagnosis, and gender when appropriate. Prior research in the older population has identified several potential reasons why individuals with multiple comorbidities are less likely to receive treatment. First, the older population is underrepresented in clinical trials of cancer therapeutics [9,11,12], leading to a lack of age-specific guidelines that oncologists rely on to make treatment decisions [42,43]. Secondly, several studies demonstrated that comorbidity could decrease treatment tolerance and increase treatment toxicity, which may indicate that the harms of specific treatments outweigh their benefits [9,10]. A recent randomized trial in lung patients with cancer showed an increase of 52% in treatment discontinuation among participants with a CCI score of two or higher [44]. A study of breast patients with cancer receiving adjuvant chemotherapy found that the odds of experiencing grade 3 or 4 treatment toxicity in patients with a CCI of one or greater was two times higher than in patients with no comorbidity [45]. Given these findings from previous studies, the actual number of patients with cancer who completed their treatment regimens could be even lower than our estimate. Finally, using multiple combinations of drugs and therapies was also associated with an increased risk of adverse events among Medicare enrollees [7], which may lead to treatment discontinuation and other serious consequences. These studies highlight the complex picture of cancer care in older patients, where risks and benefits may not be the same as for the younger population and help to explain the lower treatment rate we observed among patients with multimorbidity.

In both descriptive analysis and stratified multivariable models, our data also showed that NHW patients with multimorbidity were more likely to receive treatment than NHB and Hispanic patients with multimorbidity and NHW patients without multimorbidity were more likely to receive treatment compared to NHB without multimorbidity. These findings were consistent with previous studies [46–50]. Many studies have suggested that these treatment disparities are a primary reason for the differences in outcomes among racial groups in the United States [46]. However, treatment disparities among racial/ethnic groups are just a part of the story. Providing appropriate treatment rather than increasing the treatment rate should be the focal point of the conversation because using curative interventions that only have a modest benefit in a highly comorbid aging population could potentially decrease patients’ quality of life [14–18].

Our results by race/ethnicity suggest that NHB and Hispanic patients with multimorbidity do not experience a decline in treatment compared to the NHW patients with multimorbidity (except for breast cancer in NHB patients). This finding has not been well documented in the literature. Typically, we expect a decline in treatment rate as the patients have more comorbidities. One hypothesis could be that the NHB and Hispanic patients without multimorbidity were undertreated (compared to NHW patients), and the treatment prevalence of NHB and Hispanic patients with multimorbidity was appropriate (compared to NHW-patients). More studies are needed to confirm whether or not this is true since our current data lack the quality of life and long enough survival data to look at this issue directly.

To minimize the potential harms of both undertreatment to NH-Black and Hispanic patients without multimorbidity, we believe that a new comprehensive approach to the shared decision-making paradigm is needed since treatment decisions among older patients with cancer is heavily influenced by physician recommendation [51]. Some researchers have also suggested that specialized geriatric oncology consultation with functional status assessment could be used to screen for patients who would benefit from treatments [12,13,18,43]. However, the time-intensive nature of this approach and the lack of healthcare providers with this specialty training are significant barriers to implementing this service on a large scale [12,13,18,43]. Recent studies showed that self-administered geriatric assessment tools, which were shown to be feasible and less time-intensive, could be incorporated into clinical setting to overcome these barriers [13,52–58].

The SEER-Medicare data used in this study represents the largest sample of cancer cases among older Americans, allowing us to stratify by major cancer types, racial/ethnic groups, and the number of comorbidities. Since Medicare covered all individuals, differences in insurance coverage for cancer treatment were minimized, but differences in Medicare supplemental coverage could still affect our study results. Our study was also one of the very few to evaluate race/ethnicity in a statical interaction rather than a covariate, and our results help to shed light on differences in older cancer care among racial/ethnic groups that are often understudied, even though the older population with multiple morbidities is increasing rapidly. Although our categorization of receipts of any cancer treatment was specific to each cancer type, the current dataset did not allow us to separate between complete and guideline-concordant treatment from nonadherence or treatment dropout, and there could be overlap between curative intent and palliative intent treatment in these treatment codes. In addition, despite our ability to take several personal- and health-level variables into account, data on patients’ functional status is currently unavailable in SEER-Medicare. Moreover, our results are generalizable to this particular study period, and that new therapies are now available for these cancers, such as lung cancers, could potentially change the current treatment trend. Our approach for SES variables may still result in residual confounding, although all individuals have the same insurance (basic) coverage through Medicare. Moreover, our study only focused on the NHW, NHB, and Hispanic patients’ populations due to the small sample size of the Asian/Pacific Islander population in the dataset. We must acknowledge that we must figure out how to study these smaller but equally important minority groups who historically have experienced health disparities to ensure equitable access to cancer care. Finally, the lack of stage-specific analysis limits our ability to draw conclusions tailored to different points in the cancer care continuum, and this represents an important direction for future research.

In summary, multimorbidity increased over time among cancer patients while the receipt of treatment seems to be decreasing. Older age, a higher number of comorbidities, and advanced cancer stage were associated with lower cancer treatment among all older patients. From a clinical management perspective, multimorbidity was an independent risk factor for not receiving treatment after adjusting for other covariates, and the relationship between comorbidity and receipt of cancer treatment differed by race/ethnicity. The potential harms and underlying reasons for the racial disparities are not clear at the moment; however, we believe that specialized geriatric oncology consultation that considers complete health status as measured by comorbidity burden could improve outcomes, particularly for NHB and Hispanic patients. Further research is needed to provide a more in-depth insight into the competing health risks and the shared decision-making paradigm for the growing older population.

## Supporting information

S1 File Supplemental tables and figures. (DOCX)
