# Supplementary material for: Multimorbidity and cancer treatment among the older patients in the United States
Source: PLoS One. 2026 Jan 7;21(1):e0338721. doi: 10.1371/journal.pone.0338721 (PMC12779064; doi:10.1371/journal.pone.0338721)
Supplement: S1 File — (DOCX) [file pone.0338721.s001.docx]

**Supplemental information**

**Table A.** **Comorbidities of the study population**

|  | Breast | Colorectal | Lung | Prostate | Total |
| --- | --- | --- | --- | --- | --- |
| n (%) | 131888 (19.05) | 137414 (19.85) | 203502 (29.40) | 219355 (31.69) | 692159 (100.00) |
| Malignancy (2) | 122121 (92.59) | 109901 (79.98) | 98766 (48.53) | 208443 (95.03) | 539231 (77.91) |
| Chronic Pulmonary Disease (1) | **25818 (19.58)** | **32090 (23.35)** | **104341 (51.27)** | **41626 (18.98)** | 203875 (29.45) |
| Metastatic (6) | 9767 (7.41) | 27513 (20.02) | 104736 (51.47) | 10912 (4.97) | 152928 (22.09) |
| Diabetes without Chronic Complication (0) | 23163 (17.56) | 27135 (19.75) | 37580 (18.47) | 40036 (18.25) | 127914 (18.48) |
| Peripheral Vascular Disease (0) | 18005 (13.65) | 24231 (17.63) | 47505 (23.34) | 27451 (12.51) | 117192 (16.93) |
| Congestive Heart Failure (2) | 17240 (13.07) | 26680 (19.42) | 43399 (21.33) | 25331 (11.55) | 112650 (16.28) |
| Cerebrovascular Disease (0) | 17733 (13.45) | 22657 (16.49) | 39525 (19.42) | 27748 (12.65) | 107663 (15.55) |
| Renal Disease (1) | 5665 (4.30) | 8945 (6.51) | 14684 (7.22) | 11922 (5.44) | 41216 (5.95) |
| Diabetes with Chronic Complication (1) | 8011 (6.07) | 9392 (6.83) | 12546 (6.17) | 10820 (4.93) | 40769 (5.89) |
| Myocardial Infarction (0) | 4447 (3.37) | 8514 (6.20) | 15470 (7.60) | 11436 (5.21) | 39867 (5.76) |
| Mild Liver Disease (2) | 4206 (3.19) | 6302 (4.59) | 10387 (5.10) | 7204 (3.28) | 28099 (4.06) |
| Rheumatologic Disease (1) | 6284 (4.76) | 5429 (3.95) | 10386 (5.10) | 5795 (2.64) | 27894 (4.03) |
| Dementia (2) | 5071 (3.84) | 7106 (5.17) | 8251 (4.05) | 4631 (2.11) | 25059 (3.62) |
| Peptic Ulcer Disease (0) | 2806 (2.13) | 5861 (4.27) | 7450 (3.66) | 5213 (2.38) | 21330 (3.08) |
| Hemiplegia or Paraplegia (2) | 1940 (1.47) | 2769 (2.02) | 3947 (1.94) | 3096 (1.41) | 11752 (1.70) |
| Moderate or Severe Liver Disease (4) | 258 (0.20) | 466 (0.34) | 656 (0.32) | 325 (0.15) | 1705 (0.25) |
| HIV/AIDS (4) | 45 (0.03) | 70 (0.05) | 157 (0.08) | 176 (0.08) | 448 (0.06) |

**Table B. Univariate (unadjusted) regression model for the receipt of cancer treatment**

|  | Breast | | | Colorectal | | | Lung | | | Prostate | | |
| --- | --- | --- | --- | --- | --- | --- | --- | --- | --- | --- | --- | --- |
| VARIABLES | RR | 95% CI | p-value | RR | 95% CI | p-value | RR | 95% CI | p-value | RR | 95% CI | p-value |
| Age group |  |  |  |  |  |  |  |  |  |  |  |  |
| 65 to 70 | REF |  |  | REF |  |  | REF |  |  | REF |  |  |
| 71 to 75 | 0.993 | 0.990 - 0.996 | <0.001 | 0.998 | 0.993 - 1.004 | 0.589 | 0.938 | 0.931 - 0.945 | <0.001 | 0.964 | 0.958 - 0.970 | <0.001 |
| 76 to 80 | 0.977 | 0.973 - 0.981 | <0.001 | 0.977 | 0.971 - 0.982 | <0.001 | 0.834 | 0.827 - 0.841 | <0.001 | 0.873 | 0.867 - 0.880 | <0.001 |
| 81 or above | 0.883 | 0.879 - 0.888 | <0.001 | 0.871 | 0.865 - 0.876 | <0.001 | 0.558 | 0.551 - 0.564 | <0.001 | 0.746 | 0.739 - 0.753 | <0.001 |
| Gender |  |  |  |  |  |  |  |  |  |  |  |  |
| Female |  |  |  | REF |  |  | REF |  |  |  |  |  |
| Male |  |  |  | 0.993 | 0.989 - 0.998 | 0.004 | 1.011 | 1.004 - 1.018 | 0.001 |  |  |  |
| Race/Ethnicity |  |  |  |  |  |  |  |  |  |  |  |  |
| Non-Hispanic white | REF |  |  | REF |  |  | REF |  |  | REF |  |  |
| Non-Hispanic black | 0.932 | 0.925 - 0.940 | <0.001 | 0.923 | 0.914 - 0.932 | <0.001 | 0.895 | 0.883 - 0.907 | <0.001 | 0.883 | 0.875 - 0.892 | <0.001 |
| Hispanic | 0.986 | 0.978 - 0.994 | 0.001 | 0.988 | 0.978 - 0.998 | 0.018 | 0.947 | 0.930 - 0.965 | <0.001 | 0.989 | 0.978 - 1.000 | 0.046 |
| Year of Cancer Diagnosis |  |  |  |  |  |  |  |  |  |  |  |  |
| 1991-1995 | REF |  |  | REF |  |  | REF |  |  | REF |  |  |
| 1996-2000 | 0.995 | 0.990 - 1.001 | 0.093 | 0.999 | 0.992 - 1.006 | 0.769 | 0.984 | 0.972 - 0.997 | 0.012 | 1.043 | 1.032 - 1.053 | <0.001 |
| 2001-2005 | 0.983 | 0.978 - 0.988 | <0.001 | 1.000 | 0.993 - 1.006 | 0.952 | 0.968 | 0.957 - 0.978 | <0.001 | 1.112 | 1.103 - 1.121 | <0.001 |
| 2006-2011 | 0.972 | 0.967 - 0.976 | <0.001 | 0.938 | 0.932 - 0.944 | <0.001 | 0.965 | 0.955 - 0.975 | <0.001 | 1.058 | 1.049 - 1.067 | <0.001 |
| Indicator of lower SES |  |  |  |  |  |  |  |  |  |  |  |  |
| No | REF |  |  | REF |  |  | REF |  |  | REF |  |  |
| Yes | 0.984 | 0.981 - 0.987 | <0.001 | 0.977 | 0.972 - 0.981 | <0.001 | 0.927 | 0.920 - 0.933 | <0.001 | 0.956 | 0.951 - 0.962 | <0.001 |
| Cancer stage |  |  |  |  |  |  |  |  |  |  |  |  |
| Localized/Regional | REF |  |  | REF |  |  | REF |  |  | REF |  |  |
| Distant | 0.725 | 0.716 - 0.735 | <0.001 | 0.801 | 0.795 - 0.807 | <0.001 | 0.788 | 0.783 - 0.793 | <0.001 | 1.009 | 0.998 - 1.021 | 0.118 |
| Unknown | 0.310 | 0.296 - 0.325 | <0.001 | 0.409 | 0.398 - 0.420 | <0.001 | 0.369 | 0.360 - 0.378 | <0.001 | 0.863 | 0.856 - 0.870 | <0.001 |
| Multimorbidity |  |  |  |  |  |  |  |  |  |  |  |  |
| No | REF |  |  | REF |  |  | REF |  |  | REF |  |  |
| Yes | 0.951 | 0.947 - 0.955 | <0.001 | 0.950 | 0.945 - 0.954 | <0.001 | 0.910 | 0.904 - 0.916 | <0.001 | 0.940 | 0.934 - 0.946 | <0.001 |
| Observations | 131,888 |  |  | 137,414 |  |  | 203,502 |  |  | 219,355 |  |  |
| All RRs are unadjusted | | | | | | | | | | | | |

**Table C. Multivariable regression model for the receipt of cancer treatment using Modified Charlson Comorbidity Index.**

|  | Breast | | | Colorectal | | | Lung | | | Prostate | | |
| --- | --- | --- | --- | --- | --- | --- | --- | --- | --- | --- | --- | --- |
| VARIABLES | RR | 95% CI | p-value | RR | 95% CI | p-value | RR | 95% CI | p-value | RR | 95% CI | p-value |
| Age group |  |  |  |  |  |  |  |  |  |  |  |  |
| 65 to 70 | REF |  |  | REF |  |  | REF |  |  | REF |  |  |
| 71 to 75 | 0.996 | 0.993 - 0.999 | 0.012 | 0.999 | 0.994 - 1.005 | 0.751 | 0.943 | 0.937 - 0.950 | <0.001 | 0.965 | 0.959 - 0.970 | <0.001 |
| 76 to 80 | 0.985 | 0.981 - 0.988 | <0.001 | 0.981 | 0.976 - 0.987 | <0.001 | 0.851 | 0.844 - 0.858 | <0.001 | 0.875 | 0.869 - 0.882 | <0.001 |
| 81 or above | 0.924 | 0.920 - 0.928 | <0.001 | 0.905 | 0.900 - 0.910 | <0.001 | 0.596 | 0.590 - 0.603 | <0.001 | 0.752 | 0.745 - 0.760 | <0.001 |
| Gender |  |  |  |  |  |  |  |  |  |  |  |  |
| Female |  |  |  | REF |  |  | REF |  |  |  |  |  |
| Male |  |  |  | 0.975 | 0.971 - 0.979 | <0.001 | 0.991 | 0.985 - 0.997 | 0.003 |  |  |  |
| Race/Ethnicity |  |  |  |  |  |  |  |  |  |  |  |  |
| Non-Hispanic white | REF |  |  | REF |  |  | REF |  |  | REF |  |  |
| Non-Hispanic black | 0.955 | 0.948 - 0.961 | <0.001 | 0.944 | 0.935 - 0.952 | <0.001 | 0.916 | 0.904 - 0.928 | <0.001 | 0.883 | 0.875 - 0.892 | <0.001 |
| Hispanic | 0.983 | 0.976 - 0.990 | <0.001 | 0.990 | 0.981 - 0.999 | 0.037 | 0.976 | 0.959 - 0.993 | 0.006 | 0.986 | 0.975 - 0.997 | 0.013 |
| Year of Cancer Diagnosis |  |  |  |  |  |  |  |  |  |  |  |  |
| 1991-1995 | REF |  |  | REF |  |  | REF |  |  |  |  |  |
| 1996-2000 | 0.995 | 0.990 - 1.000 | 0.031 | 1.000 | 0.993 - 1.006 | 0.882 | 0.996 | 0.985 - 1.008 | 0.526 | 0.922 | 0.909 - 0.935 | <0.001 |
| 2001-2005 | 0.982 | 0.978 - 0.986 | <0.001 | 0.999 | 0.993 - 1.004 | 0.614 | 0.993 | 0.983 - 1.003 | 0.186 | 0.982 | 0.970 - 0.995 | 0.006 |
| 2006-2011 | 0.967 | 0.963 - 0.971 | <0.001 | 0.938 | 0.933 - 0.944 | <0.001 | 1.001 | 0.991 - 1.010 | 0.899 | 0.926 | 0.915 - 0.938 | <0.001 |
| Indicator of lower SES |  |  |  |  |  |  |  |  |  |  |  |  |
| No | REF |  |  | REF |  |  | REF |  |  | REF |  |  |
| Yes | 1.002 | 0.998 - 1.005 | 0.348 | 0.993 | 0.988 - 0.997 | 0.002 | 0.927 | 0.920 - 0.933 | <0.001 | 0.980 | 0.974 - 0.986 | <0.001 |
| Cancer stage |  |  |  |  |  |  |  |  |  |  |  |  |
| Localized/Regional | REF |  |  | REF |  |  | REF |  |  | REF |  |  |
| Distant | 0.731 | 0.722 - 0.741 | <0.001 | 0.802 | 0.796 - 0.807 | <0.001 | 0.795 | 0.790 - 0.800 | <0.001 | 1.092 | 1.080 - 1.105 | <0.001 |
| Unknown | 0.319 | 0.305 - 0.335 | <0.001 | 0.422 | 0.411 - 0.434 | <0.001 | 0.408 | 0.398 - 0.418 | <0.001 | 0.852 | 0.841 - 0.863 | <0.001 |
| Multimorbidity |  |  |  |  |  |  |  |  |  |  |  |  |
| CCI score = 0-1 | REF |  |  | REF |  |  | REF |  |  | REF |  |  |
| CCI score = 2-3 | 0.981 | 0.977 - 0.985 | <0.001 | 0.986 | 0.981 - 0.991 | <0.001 | 0.918 | 0.910 - 0.925 | <0.001 | 0.975 | 0.968 - 0.983 | <0.001 |
| CCI score = 4-5 | 0.935 | 0.925 - 0.946 | <0.001 | 0.940 | 0.930 - 0.950 | <0.001 | 0.797 | 0.785 - 0.810 | <0.001 | 0.880 | 0.863 - 0.897 | <0.001 |
| CCI score = 6-14 | 0.904 | 0.879 - 0.929 | <0.001 | 0.903 | 0.881 - 0.926 | <0.001 | 0.655 | 0.629 - 0.681 | <0.001 | 0.763 | 0.724 - 0.804 | <0.001 |
| Observations | 131,888 |  |  | 137,414 |  |  | 203,502 |  |  | 219,355 |  |  |
| All RRs are fully adjusted from the full model with age groups, gender (if applicable), race/ethnicity, year of cancer diagnosis, indicator of lower SES, cancer stage, and multimorbidity. | | | | | | | | | | | | |

**Figure A. Trends of multimorbidity over time**

**
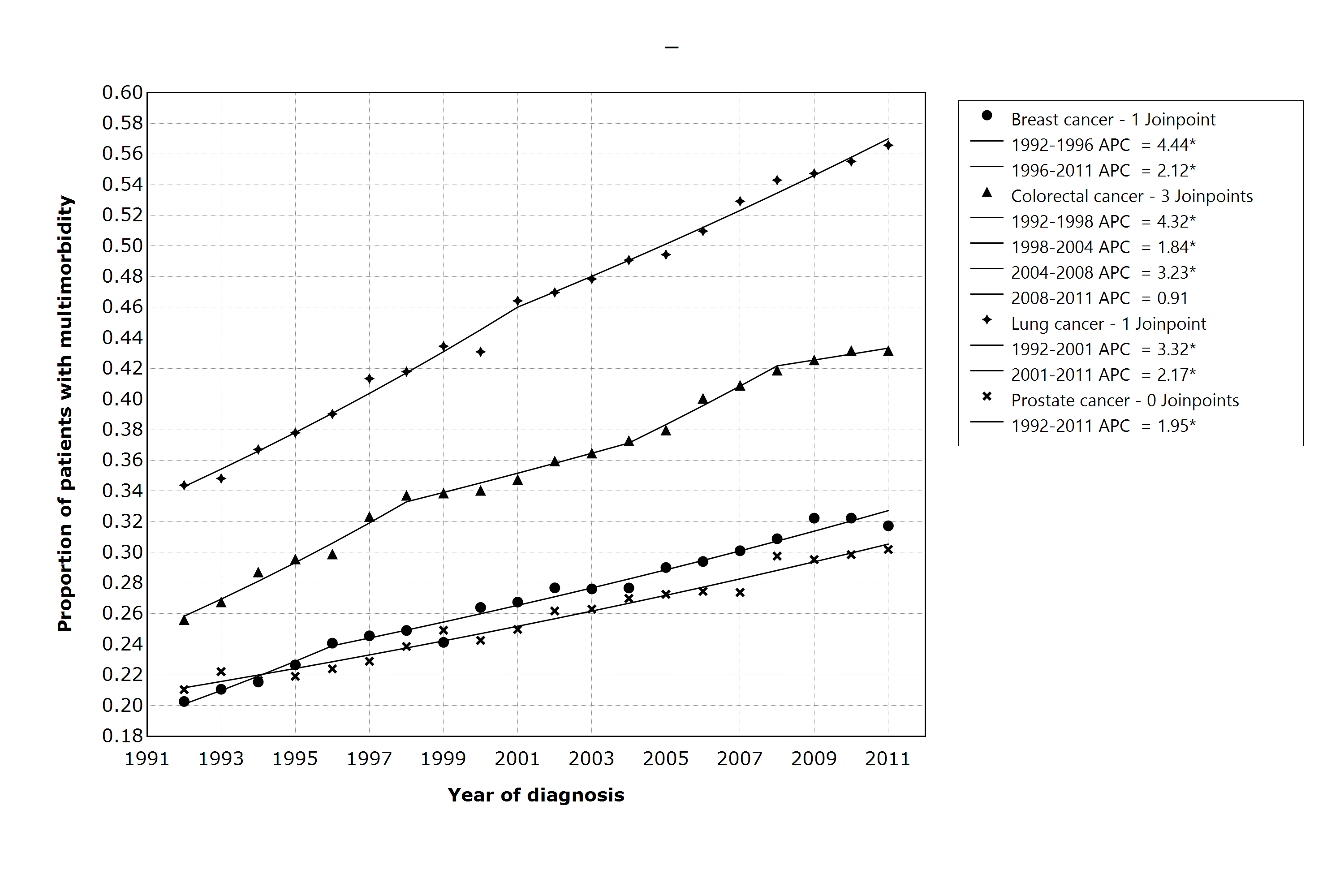
**

APC: Annual percent change of each period (%).

* indicated statiiscical significant APC change within a period

Joinpoints indicated the number of joinpoints selected by the Joinpoint statistical program

**Figure B. Trends in cancer treatment over time among patients with multimorbidity**

**
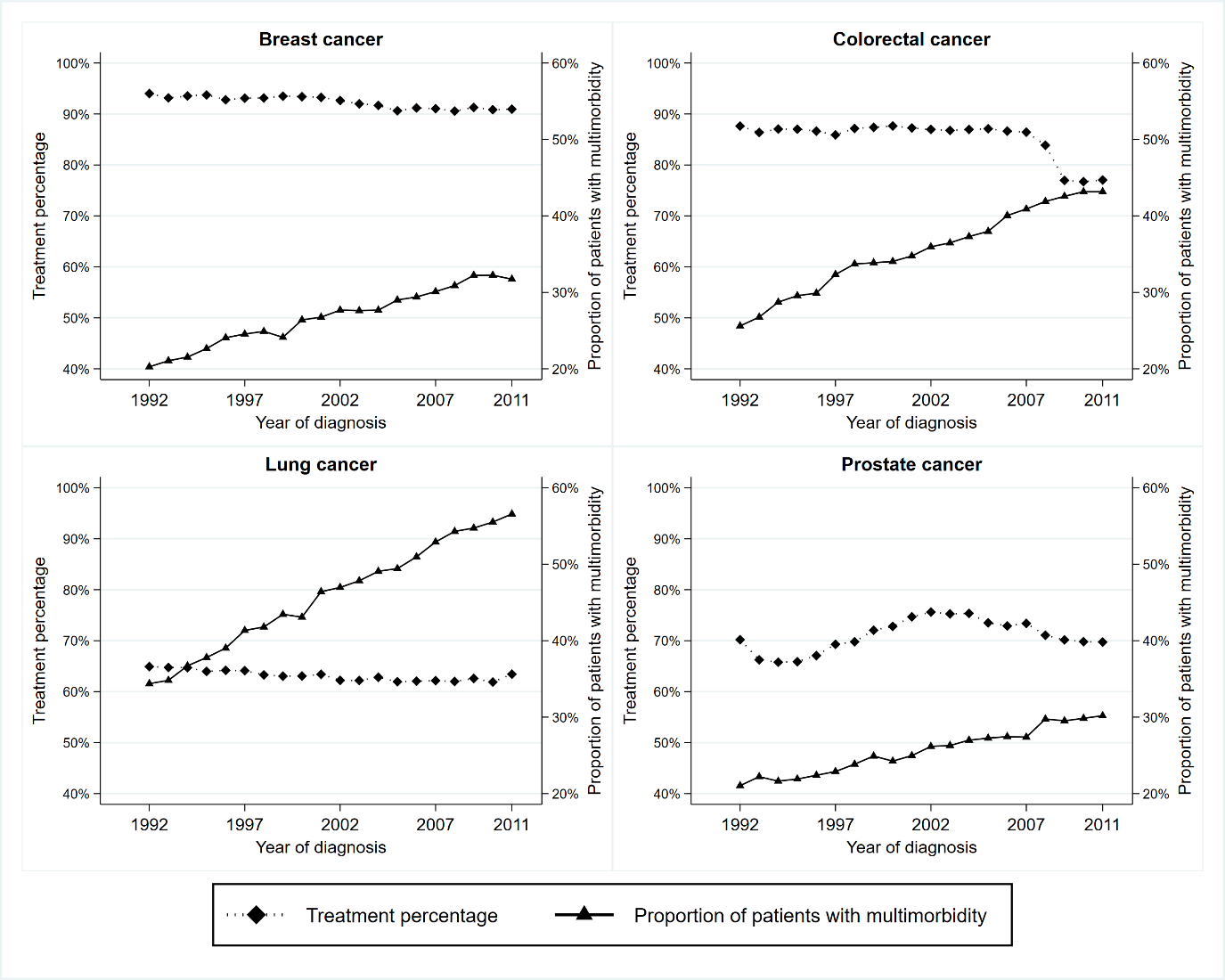
**
